# 2-(Hydroxy­meth­yl)pyridinium chloride

**DOI:** 10.1107/S1600536808034922

**Published:** 2008-10-31

**Authors:** Leigh Anna M. Ottley, Mark A. Rodriguez, Timothy J. Boyle

**Affiliations:** a Sandia National Laboratories, Advanced Materials Laboratories, 1001 University Blvd. SE, Albuquerque, NM 87106, USA; b PO Box 5800, MS 1411, Sandia National Laboratories, Albuquerque, NM 87185, USA

## Abstract

In the title molecular salt, C_6_H_8_NO^+^·Cl^−^, the packing is consolidated by N—H⋯Cl and O—H⋯Cl hydrogen bonds, resulting in the formation of [010] chains of alternating cations and anions.

## Related literature

The title compound was initially isolated by Boyle *et al.* (2008 ▶). Only the di-substituted pyridine carbonyl HCl salt has been reported previously (Fites *et al.*, 2006 ▶).
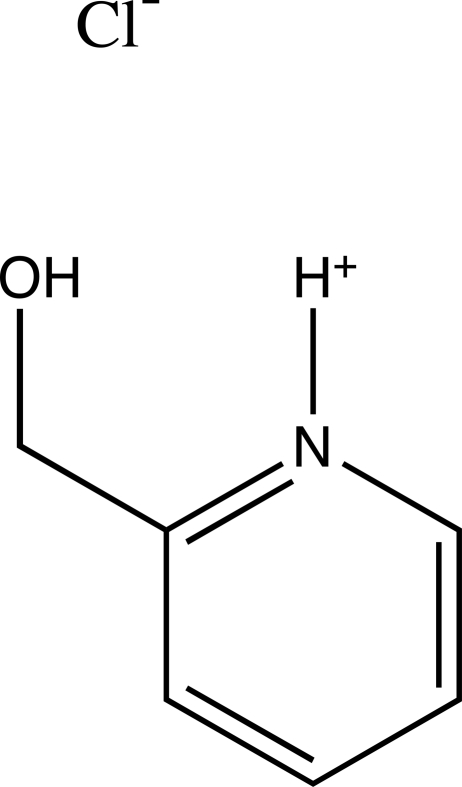


## Experimental

### 

#### Crystal data

C_6_H_8_NO^+^·Cl^−^
*M* *_r_* = 145.58
Monoclinic, 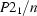

*a* = 7.0689 (9) Å
*b* = 8.0833 (11) Å
*c* = 12.1304 (16) Å
β = 102.078 (2)°
*V* = 677.79 (15) Å^3^
*Z* = 4
Mo *K*α radiation
μ = 0.47 mm^−1^
*T* = 173 (2) K
0.25 × 0.22 × 0.20 mm

#### Data collection

Bruker APEX CCD area-detector diffractometer
Absorption correction: multi-scan (*SADABS*; Sheldrick, 1999 ▶) *T*_min_ = 0.867, *T*_max_ = 0.909
4681 measured reflections
1227 independent reflections
1202 reflections with *I* > 2σ(*I*)
*R*_int_ = 0.020

#### Refinement

*R*[*F* ^2^ > 2σ(*F* ^2^)] = 0.040
*wR*(*F* ^2^) = 0.092
*S* = 1.26
1227 reflections
87 parameters
H atoms treated by a mixture of independent and constrained refinement
Δρ_max_ = 0.28 e Å^−3^
Δρ_min_ = −0.23 e Å^−3^

### 

Data collection: *SMART* (Bruker, 1998 ▶); cell refinement: *SAINT-Plus* (Bruker, 2001 ▶); data reduction: *SAINT-Plus*; program(s) used to solve structure: *SHELXS97* (Sheldrick, 2008 ▶); program(s) used to refine structure: *SHELXL97* (Sheldrick, 2008 ▶); molecular graphics: *XSHELL* (Bruker, 2000 ▶); software used to prepare material for publication: *SHELXTL* (Sheldrick, 2008 ▶).

## Supplementary Material

Crystal structure: contains datablocks I, global. DOI: 10.1107/S1600536808034922/kj2102sup1.cif

Structure factors: contains datablocks I. DOI: 10.1107/S1600536808034922/kj2102Isup2.hkl

Additional supplementary materials:  crystallographic information; 3D view; checkCIF report

## Figures and Tables

**Table 1:** Hydrogen-bond geometry (Å, °)

*D*—H⋯*A*	*D*—H	H⋯*A*	*D*⋯*A*	*D*—H⋯*A*
O1—H1⋯Cl1^i^	0.82	2.24	3.0409 (18)	167
N1—H7⋯Cl1^ii^	0.83 (3)	2.34 (3)	3.067 (2)	146 (2)

Symmetry codes: (i) 
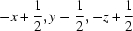
; (ii) 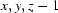.

**Fig. 1.**
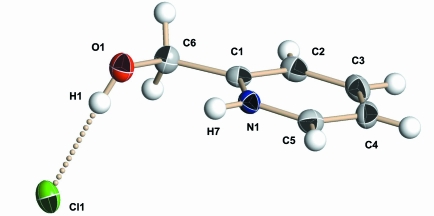
The molecular structure of the title compound, with atom labels and 50% probability atomic displacement ellipsoids for non-H atoms. The Cl atom has been translated to clarify interaction with the OH group.

**Fig. 2.**
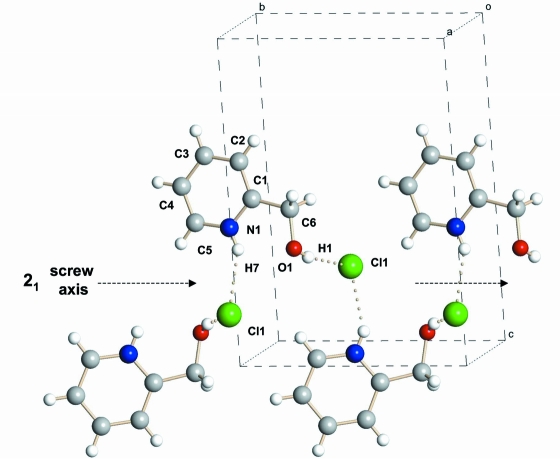
Packing of the title compound on the b-c plane illustrating the NH—Cl—OH intermolecular chain interaction which proceeds parallel to the b axis via the 21 screw axis.

## supplementary crystallographic information

### Comment

Figure 1 shows an atomic displacement ellipsoid plot of 2(hydroxymethyl)pyridinium chloride. The title compound was synthesized through the dissolution of bis(pyridine carbonoxide)titanium(dichloride), (OPy)_2_TiCl_2_, in H_2_O/HCl(5%). The synthesis was optimized by dissolving HOPy in H_2_O/HCl(5%). Fites, *et al.* (2006) reported the disubstituted salt structure which was isolated from a vanadium 2,6-pyridinedimethanol complex at low pH solutions. This is in agreement to what Boyle *et al.*(2008) found, where the title compound was isolated from low pH aqueous solutions of the titanium monosubstituted pyridinemethanol complex.

Figure 2 displays the packing arrangement of four molecules of the title compound with the Cl···H interactions that occur between adjacent molecules. The Cl interacts with the pyridinium (N1—H7···Cl1) and alcohol protons (O1—H1···Cl1), with a greater interaction observed with the alcohol, as listed in Table 1. The hydrogen bond angles for O1—H1···Cl1 and N1—H7···Cl1 are in agreement with literature angles and intermolecular interactions. In comparison, the disubstituted structure by Fites, *et al.* (2006) showed a stronger Cl binding potential with respect to the pyridinium proton (H···Cl = 2.208 Å) and a slightly weaker interaction with the alcohol (H···Cl 2.37 Å). Figure 2 also displays the pattern of H···Cl bonding throughout the unit cells. The individual molecules are related by a 2_1_ screw axis parallel to the *b* axis of the structure. The alternating interaction of the Cl between the pyridinium proton and the alcohol proton yields a intermolecular chain along the *b* axis.

### Experimental

2(Hydroxymethyl)pyridinium chloride was isolated by Boyle *et al.*(2008) through the dissolution of a titanium precursor, bis(pyridine carbonoxide)titanium(dichloride) or (OPy)_2_TiCl_2_, (where OPy = pyridine carbonoxide) in acidified water (5% of conc. HCl in water). In order to optimize the synthesis of this salt, crystal were grown *via* HOPy in acidified water (5% of conc. HCl in water). After slow evaporation, X-ray quality crystals were isolated and characterized by single-crystal X-ray, FTIR, NMR, and EA.

### Refinement

H1 (which is bound to O1 of the methanol group) was placed on ideal position, allowed to rotate around the C—O bond and refined *via* a riding model while H7 was located on difference Fourier maps and allowed to refine freely.

### Crystal data

**Table:**

C_6_H_8_NO^+^·Cl^−^	*F*(000) = 304
*M**_r_* = 145.58	*D*_x_ = 1.427 Mg m^−^^3^
Monoclinic, *P*2_1_/*n*	Mo *K*α radiation, λ = 0.71073 Å
Hall symbol: -P 2yn	Cell parameters from 200 reflections
*a* = 7.0689 (9) Å	θ = 3.1–25.2°
*b* = 8.0833 (11) Å	µ = 0.48 mm^−^^1^
*c* = 12.1304 (16) Å	*T* = 173 K
β = 102.078 (2)°	Irregular, colorless
*V* = 677.79 (15) Å^3^	0.25 × 0.22 × 0.20 mm
*Z* = 4	

### Data collection

**Table:**

Bruker APEX CCD area-detector diffractometer	1227 independent reflections
Radiation source: fine-focus sealed tube	1202 reflections with *I* > 2σ(*I*)
graphite	*R*_int_ = 0.020
φ and ω scans	θ_max_ = 25.2°, θ_min_ = 3.1°
Absorption correction: multi-scan (SADABS; Sheldrick, 1999)	*h* = −8→8
*T*_min_ = 0.867, *T*_max_ = 0.909	*k* = −9→9
4681 measured reflections	*l* = −14→13

### Refinement

**Table:**

Refinement on *F*^2^	Primary atom site location: structure-invariant direct methods
Least-squares matrix: full	Secondary atom site location: difference Fourier map
*R*[*F*^2^ > 2σ(*F*^2^)] = 0.040	Hydrogen site location: inferred from neighbouring sites
*wR*(*F*^2^) = 0.093	H atoms treated by a mixture of independent and constrained refinement
*S* = 1.26	*w* = 1/[σ^2^(*F*_o_^2^) + (0.0271*P*)^2^ + 0.6606*P*] where *P* = (*F*_o_^2^ + 2*F*_c_^2^)/3
1227 reflections	(Δ/σ)_max_ < 0.001
87 parameters	Δρ_max_ = 0.28 e Å^−^^3^
0 restraints	Δρ_min_ = −0.23 e Å^−^^3^

### Special details

**Table:**

Geometry. All e.s.d.'s (except the e.s.d. in the dihedral angle between two l.s. planes) are estimated using the full covariance matrix. The cell e.s.d.'s are taken into account individually in the estimation of e.s.d.'s in distances, angles and torsion angles; correlations between e.s.d.'s in cell parameters are only used when they are defined by crystal symmetry. An approximate (isotropic) treatment of cell e.s.d.'s is used for estimating e.s.d.'s involving l.s. planes.
Refinement. Refinement of *F*^2^ against ALL reflections. The weighted *R*-factor *wR* and goodness of fit *S* are based on *F*^2^, conventional *R*-factors *R* are based on *F*, with *F* set to zero for negative *F*^2^. The threshold expression of *F*^2^ > σ(*F*^2^) is used only for calculating *R*-factors(gt) *etc*. and is not relevant to the choice of reflections for refinement. *R*-factors based on *F*^2^ are statistically about twice as large as those based on *F*, and *R*- factors based on ALL data will be even larger.

### Fractional atomic coordinates and isotropic or equivalent isotropic displacement parameters (Å^2^)

**Table:**

	*x*	*y*	*z*	*U*_iso_*/*U*_eq_	
Cl1	0.10702 (9)	0.46272 (7)	0.68760 (4)	0.0311 (2)	
N1	0.2053 (3)	0.4526 (2)	−0.05399 (16)	0.0213 (4)	
O1	0.0732 (2)	0.1503 (2)	−0.10825 (13)	0.0292 (4)	
H1	0.1498	0.1040	−0.1403	0.044*	
C1	0.2214 (3)	0.3472 (3)	0.03263 (18)	0.0220 (5)	
C4	0.3390 (3)	0.6739 (3)	0.0620 (2)	0.0299 (5)	
H4	0.3773	0.7840	0.0715	0.036*	
C5	0.2628 (3)	0.6116 (3)	−0.04288 (19)	0.0264 (5)	
H5	0.2509	0.6789	−0.1062	0.032*	
C3	0.3576 (3)	0.5680 (3)	0.1541 (2)	0.0312 (6)	
H3	0.4104	0.6074	0.2260	0.037*	
C2	0.2989 (3)	0.4061 (3)	0.14015 (19)	0.0271 (5)	
H2	0.3109	0.3364	0.2022	0.033*	
C6	0.1592 (3)	0.1715 (3)	0.00685 (18)	0.0273 (5)	
H6A	0.2708	0.0993	0.0266	0.033*	
H6B	0.0674	0.1402	0.0524	0.033*	
H7	0.165 (4)	0.414 (3)	−0.118 (2)	0.027 (7)*	

### Atomic displacement parameters (Å^2^)

**Table:**

	*U*^11^	*U*^22^	*U*^33^	*U*^12^	*U*^13^	*U*^23^
Cl1	0.0410 (4)	0.0293 (3)	0.0204 (3)	−0.0037 (2)	0.0005 (2)	0.0035 (2)
N1	0.0213 (9)	0.0237 (10)	0.0181 (9)	0.0011 (8)	0.0023 (7)	−0.0017 (8)
O1	0.0326 (9)	0.0303 (9)	0.0229 (8)	−0.0027 (7)	0.0015 (7)	−0.0028 (7)
C1	0.0194 (11)	0.0260 (11)	0.0209 (11)	0.0026 (9)	0.0050 (8)	0.0030 (9)
C4	0.0264 (12)	0.0236 (12)	0.0391 (14)	−0.0003 (10)	0.0052 (10)	−0.0071 (10)
C5	0.0266 (12)	0.0228 (12)	0.0305 (12)	0.0038 (9)	0.0076 (9)	0.0029 (10)
C3	0.0271 (12)	0.0382 (14)	0.0266 (12)	0.0034 (11)	0.0018 (9)	−0.0102 (11)
C2	0.0273 (12)	0.0340 (13)	0.0197 (11)	0.0033 (10)	0.0038 (9)	0.0012 (10)
C6	0.0323 (13)	0.0260 (12)	0.0223 (11)	−0.0020 (10)	0.0026 (9)	0.0028 (9)

### Geometric parameters (Å, °)

**Table:**

N1—C1	1.339 (3)	C4—C3	1.392 (4)
N1—C5	1.347 (3)	C4—H4	0.9300
N1—H7	0.83 (3)	C5—H5	0.9300
O1—C6	1.412 (3)	C3—C2	1.372 (3)
O1—H1	0.8200	C3—H3	0.9300
C1—C2	1.389 (3)	C2—H2	0.9300
C1—C6	1.500 (3)	C6—H6A	0.9700
C4—C5	1.370 (3)	C6—H6B	0.9700
			
C1—N1—C5	123.7 (2)	C2—C3—C4	120.8 (2)
C1—N1—H7	116.8 (18)	C2—C3—H3	119.6
C5—N1—H7	119.3 (18)	C4—C3—H3	119.6
C6—O1—H1	109.5	C3—C2—C1	119.5 (2)
N1—C1—C2	118.1 (2)	C3—C2—H2	120.2
N1—C1—C6	117.76 (19)	C1—C2—H2	120.2
C2—C1—C6	124.2 (2)	O1—C6—C1	111.60 (18)
C5—C4—C3	118.2 (2)	O1—C6—H6A	109.3
C5—C4—H4	120.9	C1—C6—H6A	109.3
C3—C4—H4	120.9	O1—C6—H6B	109.3
N1—C5—C4	119.7 (2)	C1—C6—H6B	109.3
N1—C5—H5	120.1	H6A—C6—H6B	108.0
C4—C5—H5	120.1		

### Hydrogen-bond geometry (Å, °)

**Table:**

*D*—H···*A*	*D*—H	H···*A*	*D*···*A*	*D*—H···*A*
O1—H1···Cl1^i^	0.82	2.24	3.0409 (18)	167
N1—H7···Cl1^ii^	0.83 (3)	2.34 (3)	3.067 (2)	146 (2)

Symmetry codes: (i) −*x*+1/2, *y*−1/2, −*z*+1/2; (ii) *x*, *y*, *z*−1.
